# The impact of temperature on vascular function in connection with vascular laser treatment

**DOI:** 10.1007/s10103-024-04070-7

**Published:** 2024-05-04

**Authors:** M. Doppegieter, T.G. van Leeuwen, M.C.G. Aalders, J. de Vos, E.T. van Bavel, E.N.T.P Bakker

**Affiliations:** 1 Amsterdam UMC location University of Amsterdam, Biomedical Engineering and Physics, Meibergdreef 9, Amsterdam, the Netherlands; 2 Amsterdam Cardiovascular Sciences, Microcirculation, Amsterdam, the Netherlands; 3 Amsterdam Cardiovascular Sciences, Heart Failure and Arrhythmias, Amsterdam, the Netherlands; 4 Amsterdam Cardiovascular Sciences, Atherosclerosis & Ischemic Syndromes, Amsterdam, the Netherlands; 5 Cancer Center Amsterdam, Imaging and Biomarkers, Amsterdam, the Netherlands; 6 Co van Ledden Hulsebosch Center, University of Amsterdam, Science Park, 904 Amsterdam, The Netherlands; 7 Amsterdam Public Health, Personalized medicine, Amsterdam, the Netherlands; 8 Amsterdam Neuroscience, Neurovascular Disorders, Amsterdam, the Netherlands

**Keywords:** Hyperthermia, Blood vessels, Wire myography, Vascular nerves, Smooth muscle cells, Endothelial cells

## Abstract

Pulsed dye lasers are used effectively in the treatment of psoriasis with long remission time and limited side effects. It is, however, not completely understood which biological processes underlie its favorable outcome. Pulsed dye laser treatment at 585-595 nm targets hemoglobin in the blood, inducing local hyperthermia in surrounding blood vessels and adjacent tissues. While the impact of destructive temperatures on blood vessels has been well studied, the effects of lower temperatures on the function of several cell types within the blood vessel wall and its periphery are not known. The aim of our study is to assess the functionality of isolated blood vessels after exposure to moderate hyperthermia (45 to 60°C) by evaluating the function of endothelial cells, smooth muscle cells, and vascular nerves. We measured blood vessel functionality of rat mesenteric arteries (*n*=19) by measuring vascular contraction and relaxation before and after heating vessels in a wire myograph. To this end, we elicited vascular contraction by addition of either high potassium solution or the thromboxane analogue U46619 to stimulate smooth muscle cells, and electrical field stimulation (EFS) to stimulate nerves. For measurement of endothelium-dependent relaxation, we used methacholine. Each vessel was exposed to one temperature in the range of 45-60°C for 30 seconds and a relative change in functional response after hyperthermia was determined by comparison with the response per stimulus before heating. Non-linear regression was used to fit our dataset to obtain the temperature needed to reduce blood vessel function by 50% (Half maximal effective temperature, ET50). Our findings demonstrate a substantial decrease in relative functional response for all three cell types following exposure to 55°C-60°C. There was no significant difference between the ET50 values of the different cell types, which was between 55.9°C and 56.9°C (*P*>0.05). Our data show that blood vessel functionality decreases significantly when exposed to temperatures between 55°C-60°C for 30 seconds. The results show functionality of endothelial cells, smooth muscle cells, and vascular nerves is similarly impaired. These results help to understand the biological effects of hyperthermia and may aid in tailoring laser and light strategies for selective photothermolysis that contribute to disease modification of psoriasis after pulsed dye laser treatment.

**Supplementary Information:**

The online version contains supplementary material available at 10.1007/s10103-024-04070-7.

## Introduction

Lasers are used for the treatment of vascular lesions such as telangiectasia, spider veins, psoriasis, and port-wine stains [1, 2]. Various types of lasers can effectively heat blood vessels in the skin and their working mechanism is based on the selective light absorption by (oxy)hemoglobin in blood vessels inducing thermal coagulation and vascular destruction. In the case of psoriasis, treatment by pulsed dye laser (PDL) irradiation (commonly used at 585-595 nm, 0.45 or 1.5 ms between 6-9 J/cm^2^ ) has shown its clinical value because of limited side effects and surprisingly long (~1 year) remission [3–7]. Yet, its mechanism remains poorly understood. It was first believed that the appearance of purpura shortly after a laser pulse was an indicator of sufficient laser power and thus for achieving clinical improvement of the psoriasis lesion [6, 7]. However, as devices such as intense pulsed light (IPL) also show clinical improvement in psoriasis, bursting of the vessel may not be necessary [8]. This finding suggests that despite the correlation between psoriasis improvement and reduced microvascular density [9], destruction of the blood vessels may not be necessary for the clinical remission of psoriasis. It should be noted in this context, that PDL selectively targets the red blood cells, as the 585-595 nm laser light is absorbed by hemoglobin [10].

Increasing evidence suggests that psoriasis is the consequence of neurogenic inflammation [11–14]. We recently published a review wherein we stated the hypothesis that pulsed dye laser (PDL) treatment may damage the (peri)vascular nerves through dissipating heat, which could explain the underlying mechanism for long-term remission after PDL treatment in psoriasis [15]. Long-term nerve injury due to lasers is furthermore supported by the case reports on ocular damage inflicting pain or loss of sight after accidental exposure to laser light, that is often irreversible and requires surgical procedures [16]. Hyperthermia-induced nerve damage is hard to assess, as nerve morphology in microscopic images does not portray the activity of such nerves. Understanding the effects of moderate hyperthermia on blood vessels and their surrounding vascular nerves can provide insight into the overall biological effect of laser treatment. Therefore, this study aimed to assess the functionality of isolated blood vessels after exposure to moderate hyperthermia (45 to 60°C) by evaluating the response to cell-specific stimuli for endothelial cells, smooth muscle cells, and vascular nerves. The hypothesis is that the sensitivity to thermal damage differs between cell types.

## Material and methods

### Animals

**Fig. 1:**
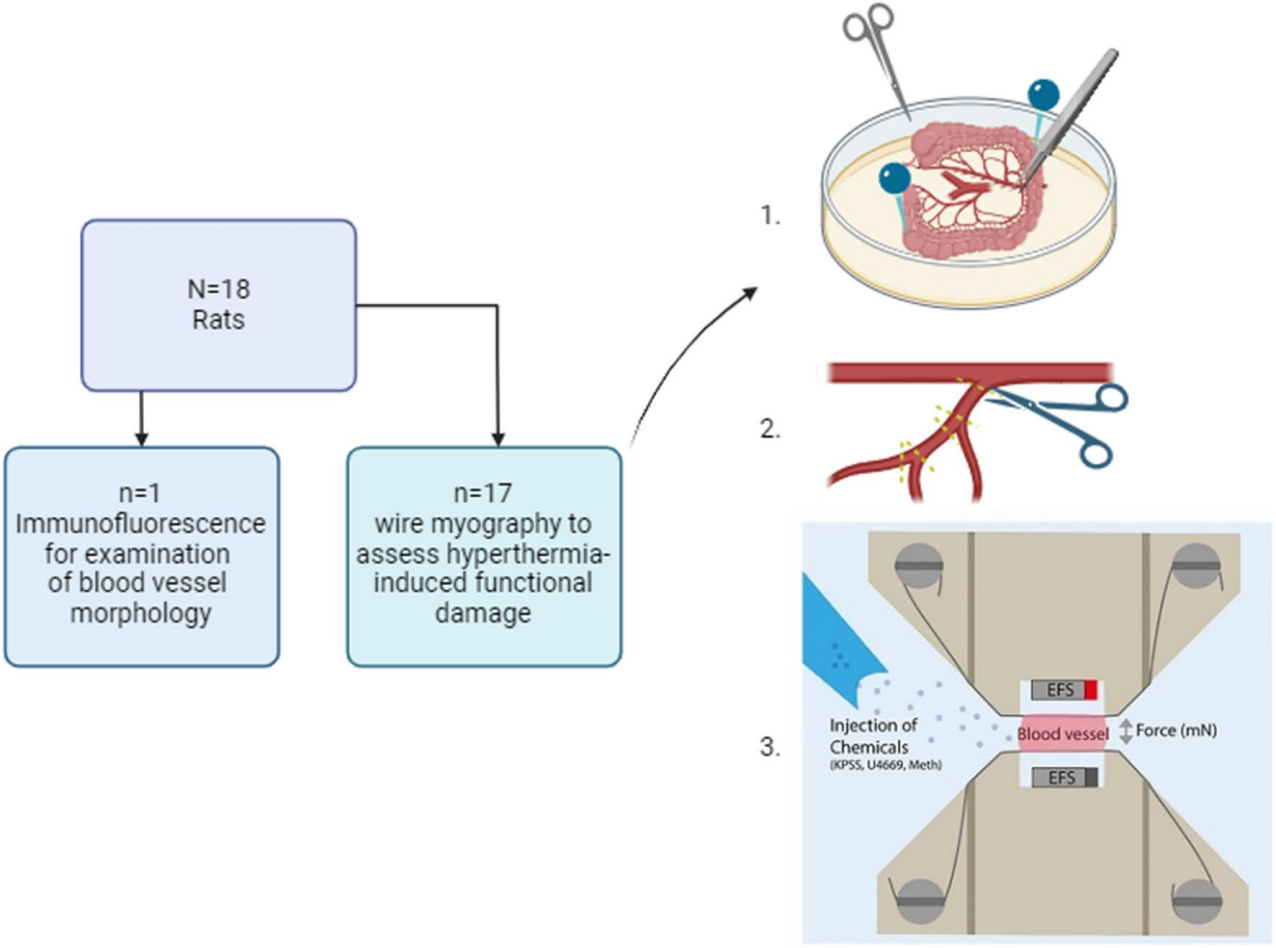
Diagram of the distribution of rats and illustration of the technical procedure for the wire-myograph experiment

Eighteen Lewis rats (9 female, 9 male) aged 12 to 16 weeks, weighing 270 ± 55 g were deeply anesthetized using isoflurane (isoflutek 1000 mg/g, Karizoo, Barcelona, Spain) and after subsequent decapitation, second and/or third-order mesenteric arteries, each approximately2 mm in length, were dissected with dissection tools (student dissection kit 504167, world precision instruments, WPI-Europe, Friedberg, Germany) and isolated from the rat mesenteric vascular bed (Fig. 1). All procedures were conducted in accordance with the guidelines of the National Institute of Health (NIH) for the care and use of laboratory animals and institutional protocols.

### Immunohistochemistry

The presence and morphological integrity of endothelial cells, smooth muscle cells, and vascular nerves were confirmed by fluorescence immunohistochemistry. One rat was anesthetized and infused with Lectin conjugated to AF594 (DL-1177, Vectorlabs, Vector Laboratories, Newark, United States)) to stain for endothelial cells and then fixed with PFA to prevent a collapse of the blood vessels. Vessel segments were fixed in Zamboni Fixative (263-01991, Bio-connect,FUJIFILM Wako, Neuss, Germany) for 24 h at 4°C and then washed three times in dPBS (PBS-1A, Capricorn Scientific, Capricorn Scientific, Germany). Blocking of non-specific antigen binding was performed with 4% normal goat serum (X090710-8, Agilent, Agilent technologies, Amstelveen, The Netherlands) and 1% Triton X-100 (9036-19-5, BOOM, Merck, The Netherlands) in PBS for 2 h at room temperature. Then, vessels were incubated with rabbit anti-rat protein gene product 9.5 (PGP9.5) 1:500 (ab108986, Abcam, Cambridge, United Kingdom), Neuronal peptide Y (NPY-EPR21877, ab221145, abcam, Cambridge, United Kingdom,), or calcitonin gene-related peptide (CGRP-4901, ab81887, abcam, Cambridge, United Kingdom) overnight at 4°C. Primary antibodies were diluted in a 1:1 mixture of PBS and blocking solution. Vessels were washed thrice with PBS and concordantly incubated with goat anti-rabbit cy3 (111-165-144, Brunschwig, Jackson immune Research, Cambridgeshire, United Kingdom), at 1:800 dilution in PBS for 2 h at RT. Vessels were rinsed in milliQ (CDUFBI001 Q-Pod, Biopak, Merck, The Netherlands) and mounted on a glass slide in DAPI vectashield (H-1200-10, Vectashield, Vector Laboratories, Newark, United States). A 3D set of images were obtained with a Leica SPM-8X Confocal Microscope (DLS lightsheet, Leica, Leica Microsystems BV, Amsterdam, The Netherlands) equipped with a 40x/1.3 OIL objective.

### Wire myography

The isolated arteries were positioned in a wire-myograph setup (620M, Danish Myo Technology, DMT, Aarhus, Denmark) to measure the contractile force generated by the artery under different conditions. Rat mesenteric first and second-order arteries were used with a mean diameter of 360 ± 60 μm and a mean vessel length of 1.9 ± 0.1 mm. Vessels were exposed to various temperatures and subsequently further stimulated using either agonists or transversal electrical field stimulation (EFS) by electrodes next to the blood vessel. The wire-myograph was also equipped with an in-house assembled device using K-type thermocouples (621-2158, RS, RS Components BV, Haarlem, The Netherlands) that measured temperature fluctuations in each vessel chamber, calibrated for a temperature range of 20°C to 70°C.

At the start of the experiment, vessels were equilibrated to 37°C, and the vessel was pre-stretched in calcium-free MOPS buffer (for buffer composition see supplemental Table 1). Calcium-free conditions were used here to prevent smooth muscle contraction. This procedure was used to obtain the optimal level of pre-stretch for each vessel, which is at 90% of the diameter at 100 mmHg. At this level of pre-stretch vascular responses are maximal. [17, 18] The result of this procedure is a passive force of the vessel around 3 to 4 mN. Then, vessels were acclimatized for ten minutes in 37°C physiological salt solution (PSS) (see components in supplemental Table 1) and the chamber was aerated with a gas mixture of 95% air and 5% CO_2_ (UN1956, 1470110, Lindegas, The Netherlands) to maintain metabolic activity of the cells. Vessels were then exposed shortly to 125 mmol / L potassium solution (KPSS, see supplemental Table 1) to test for viability, rinsed thrice with PSS, and rested for five minutes. Vessels demonstrating a contractile force of less than 10 mN to the non-receptor-mediated stimulation of smooth muscle cells by KPSS were considered to be damaged and excluded from the experiment. The vessels were then contracted with 1 x 10^-5^ μmol U46619 (L-thromboxane A_2_ agonist, D8174, Sigma Aldrich, Amsterdam, The Netherlands). The contracted state upon exposure to U46619 was then used to assess vascular relaxation through the addition of 1 x 10^-5^ mol/L methacholine (A2251, Sigma Aldrich, Amsterdam, The Netherlands), which is an endothelium-dependent vasorelaxing agent (non-selective muscarinic receptor agonist). The chamber was washed thrice with PSS and the vessels were rested for five minutes. Lastly, electrical field stimulation was used to stimulate nerve endings to initiate smooth muscle cell-mediated contraction. EFS potentially stimulates both nerves and smooth muscle cells at the same time, hampering the possibility of assessing the involvement of nerves in the response. Therefore, the frequency of EFS that directly induces smooth muscle contraction was determined by using a nerve blocker (3 μM TTX, HB1035, HelloBio, Dunshaughlin, Ireland ). Blocking nerve function while applying EFS showed that smooth muscle contraction appears at 32 Hz and higher frequencies (Supplemental Fig. 2). Therefore, our experiments were performed in the nerve-specific range of 0.5 Hz – 16 Hz. In more detail, the frequency consisted of a range of 0.5, 1, 2, 4, 8, and 16 Hz, each with a pulse duration of 0.4 ms and 40 mA, and pulse lengths of 10 sec with 1-sec breaks between incrementing frequencies (0.5Hz to 1 Hz, 1Hz to 2Hz, etc). Vessels were stimulated by three sets of EFS with a 120-second rest in between. Repeating the EFS three times allowed the blood vessel to reach the maximal contraction. Vessels were then rested for 10 minutes and subsequently exposed to heated PSS in the range of 42°C – 62°C, or kept at 37°C (control). The heated PSS was administered into the chamber by a pre-warmed plastic syringe. After 30 seconds, the PSS was replaced with PSS at 37°C, and vessels were rested for 5 minutes. Then, the protocol was repeated (KPSS, U46619, methacholine, EFS). Maximum values of individual vessels were compared before and after hyperthermia.

### Statistics

Maximal contraction force was retrieved using the max-min function in Chart 5 (V5.5.6, ADInstruments, Oxford, United Kingdom). Maximal force (in mN) after heating was divided by the maximal force before heating, yielding a ratio for each response that was used to assess hyperthermia-induced functional damage. Averaging these ratios from individual vessels resulted in a mean ± SEM of relative response R as shown in Fig. 4. A non-linear four-parameter fit of relative response *R*(*T*) forced through zero was applied to the individual data points with *R*_*max*_, *ET*50 *and γ* as fitting parameters to find the temperature at which 50% of the vessel functionality was lost (Effective Temperature, ET50): $R(T)=\frac{R_{max}}{\left(1+{10}^{\left( LD50-T\right)\bullet \gamma}\right)}$ where *R*_*max*_ is the maximum response, and *γ* the decrease in relative response (contraction or vasorelaxation) per °C increase in temperature. To compare the ET50 for the four stimuli we performed a Least Squares regression analysis with multiple comparison testing and Bonferroni correction, *α* = 0.05 . We compared all three cell types by testing the ET50 values of KPSS vs Methacholine, KPSS vs EFS, and EFS vs Methacholine. Here, the cut-off value for statistical significance was adapted for three comparisons at 0.05 / 3 = 0.017. ET50 values are shown with their symmetric 95% confidence interval (ET50 ± CI95). All statistical analyses and data visualization were performed using GraphPad Prism Software v.8.3 (Prism, Graphpad, Graphpad Software, Boston, Massachusetts, United States).

## Results

### Morphology of the rat mesenteric artery

**Fig. 2:**
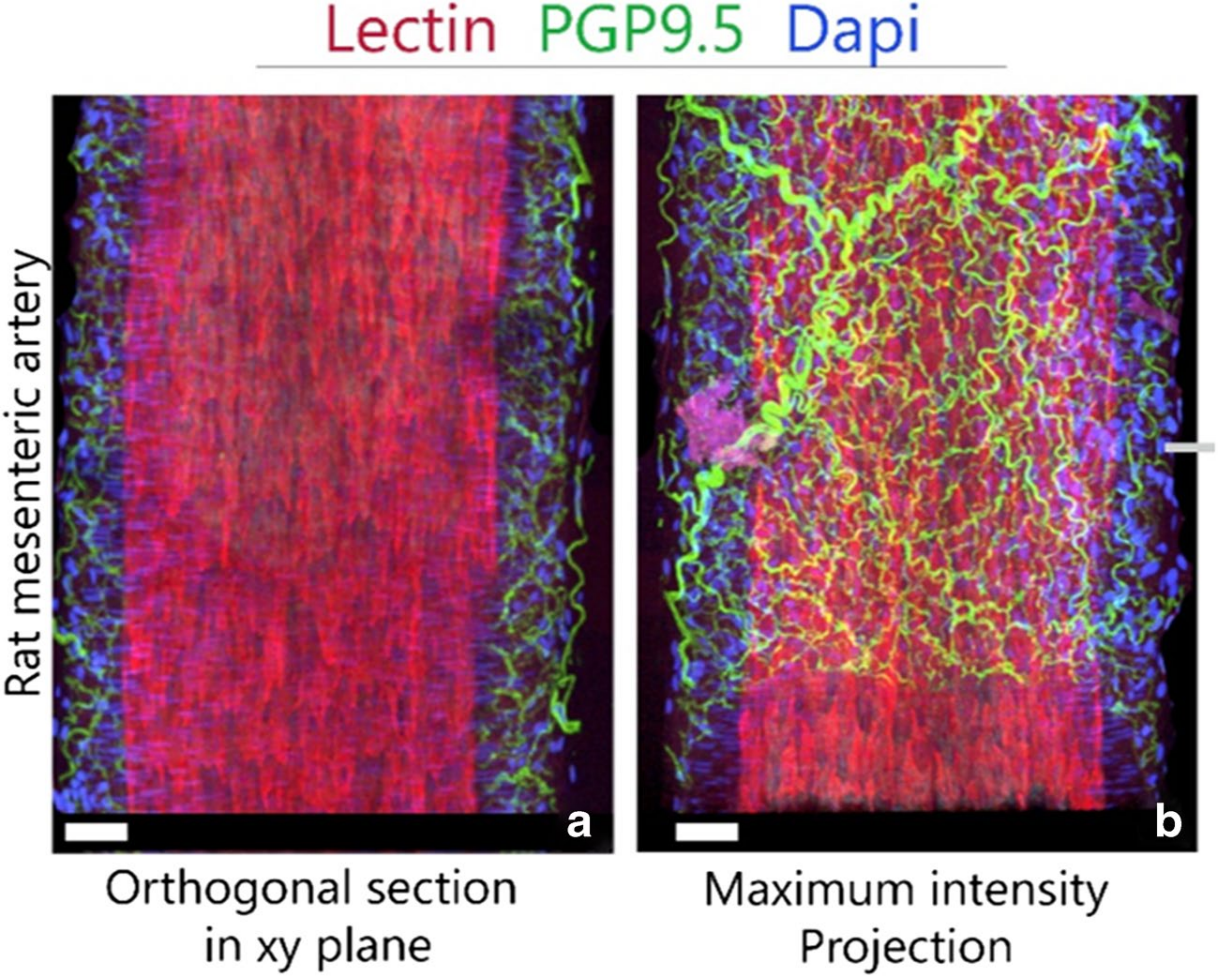
Confocal images (**A**. and **B**.) of a rat mesenteric artery with endothelium (lectin, red), nuclei of the smooth muscle cells (dapi, blue), and nerves (PGP-9.5, green). **A**. shows a mid-section of the artery segment, **B**. shows the maximum intensity projection of the z-stack. Scale bars indicate 100 μm

Immunohistochemistry confirmed the presence of endothelial cells, smooth muscle cells, and vascular nerves within the rat mesenteric artery, showing the morphology of the vessel wall and its integrity after dissection. As depicted in Fig. 2, the inner lining of the blood vessel is formed by endothelial cells (marked in red, lectin), followed by a layer of smooth muscle cells (marked in blue, α-SMA), and a plexus of vascular nerves (marked in green, PGP-9.5) (supplementary video 1). A closer examination of the vascular nerve plexus revealed a double-layered structure. The first layer consisted of thinner, sympathetic, nerve fibers positive for neuropeptide Y (NPY), that were nestled between the smooth muscle cells. The outer layer of the nerve plexus contained thicker and less dense (sensory) nerve fibers positive for calcitonin gene-related peptide (CGRP) (Supplementary Fig. 1).

### Blood vessel functionality before and after hyperthermia

**Fig. 3:**
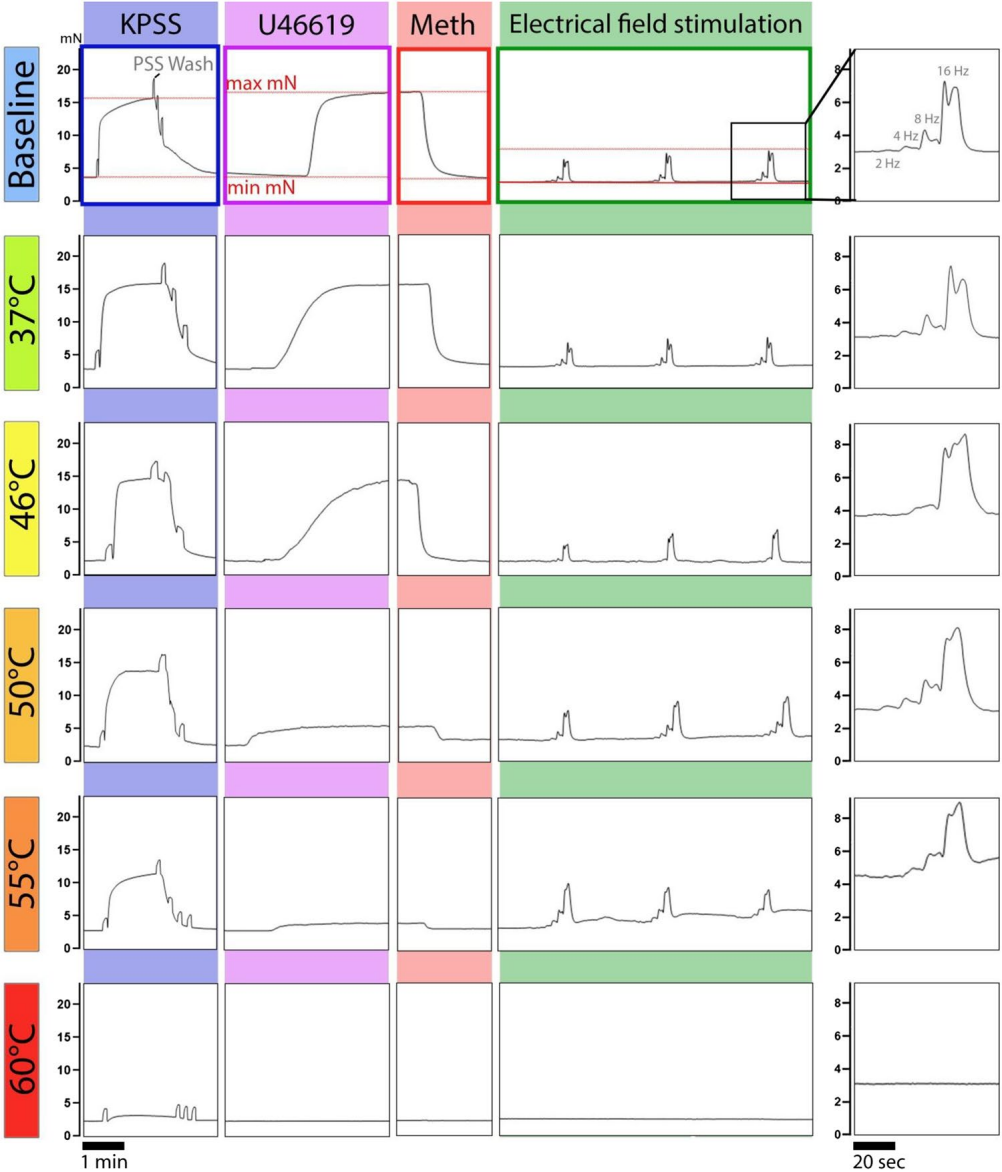
Example of raw wire myography data showing the force in milliNewton (mN) produced by the arteries before and after temperature exposure that lasted 30 seconds. Baseline values upon stimuli were recorded for all vessels after which they were exposed to a designated temperature, after which stimuli were repeated. Active force data was obtained by subtracting the baseline response from the maximum force value (indicated by red lines). The plots indicate that KPSS maintained reproducible contraction at around 15 mN for temperatures up to 50°C. In most cases, contraction by U46619 shows a strong decline at temperatures higher than 46°C. For electrical field stimulation (EFS) incomplete relaxation in-between stimulation was observed at 55°C and the complete absence of EFS-contraction occurred at 60°C

Fig. 3 shows the force produced by the vessel upon stimulation with KPSS, U46619, Methacholine, and EFS in time and as a function of temperature. Each blood vessel was exposed only once to a specific temperature. The first frame in Fig. 3 shows that KPSS stimulation resulted in the strongest and most consistent contraction among all stimuli. The KPSS was then washed out thrice, resulting in three small increases in force due to the flow of liquid within the measurement chamber. The second frame in Fig. 3 shows the contraction as a response to U46619. This pre-contracted state was then used to assess the endothelial-mediated vasorelaxing response to methacholine resulting in a decrease in force (mN) measured. Finally, the last frame shows the effect of nerve stimulation and consecutive vasocontraction during EFS. The EFS-frame of the vessel exposed to 55°C shows that there is incomplete relaxation after EFS stimulation. For all stimuli the response was decreased after ~55°C and abolished at ~60°C.

### Vascular contraction

**Fig. 4:**
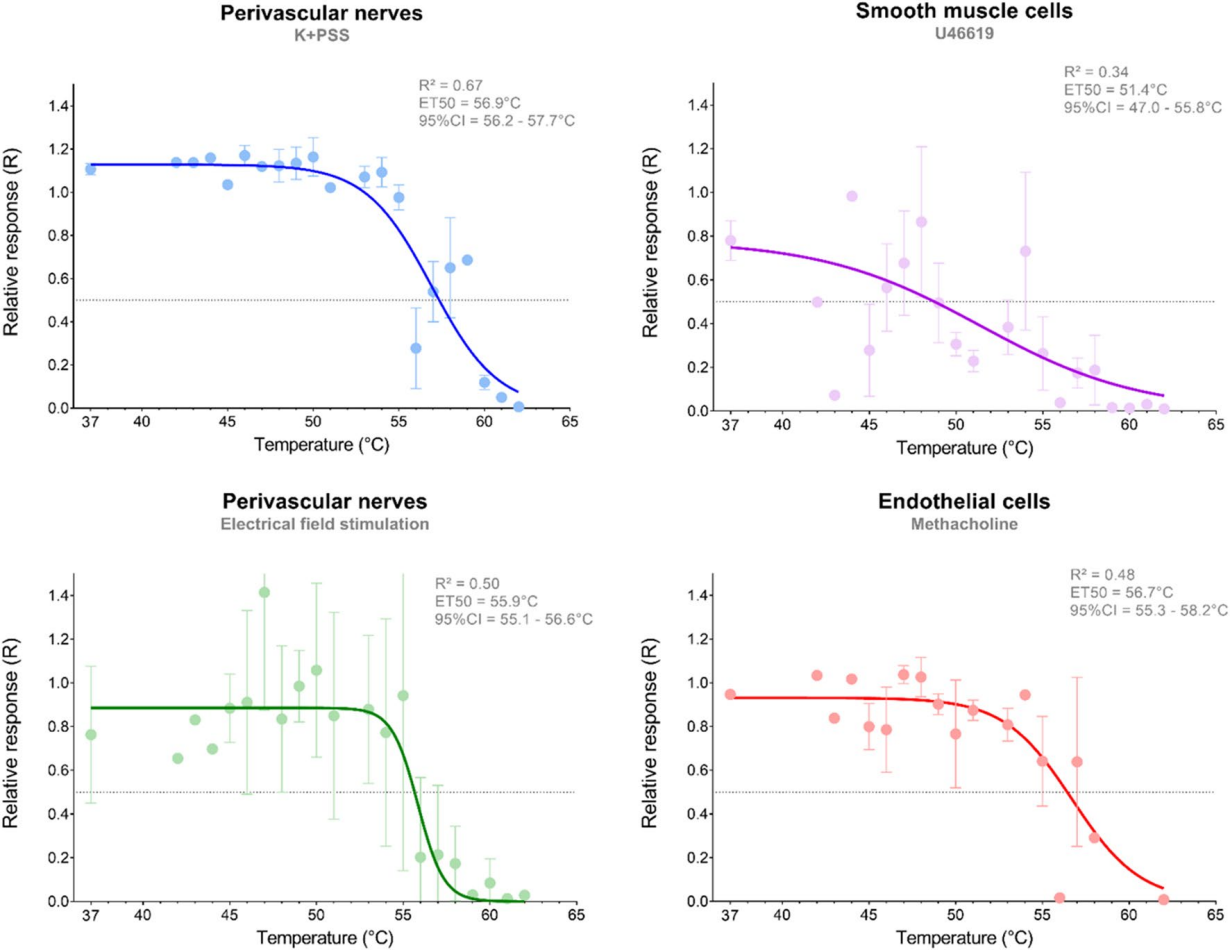
Impaired functionality of rat mesenteric arteries after hyperthermia. The figure displays the least squares regression fit (line), with each point representing the mean ± SEM of the ratio before and after heating. Control groups were subjected to 37°C conditions. The graphs show a grey-dotted line at 0.5 at which the intersection represents the ET50: The temperature at which vessel function is decreased by 50%. The goodness of fit (R2) and the 95% confidence intervals for the ET50 are indicated for each stimulus. The graphs represent distinct stimuli: KPSS, U46619, methacholine, and electrical field stimulation, each chosen to target specific cell types within the artery

The non-receptor-mediated contractile force induced by KPSS was maintained for the first part of the temperature range but noticeably decreased after exposure to 55°C (Fig. 4). The temperature at which the KPSS response was reduced by 50% (ET50) was calculated at 56.9°C (56.2-57.7°C), with a goodness of fit (R^2^) of 67% (Fig. 4, supplemental Table 2). Receptor-mediated stimulation of smooth muscle cells by U46619 led to a decline in contractile force after exposure to temperatures around 50°C. The ET50 for U46619-induced contraction was calculated to be 51.5°C (47.0-55.8°C), with an R^2^ of 34%. In some instances, the loss of contraction occurred even before heating was applied or upon exposure to 37°C. Consequently, this inconsistency in response patterns affected the 95% confidence interval for the ET50 of the U46619 reaction, which ranged from 47.0°C to 55.8°C.

The maximal response to nerve-regulated smooth muscle cell contraction by EFS was consistently observed to be at its maximum at 16 Hz and exhibited a decline in response after exposure to 56°C – 60°C. In comparison to KPSS, EFS-induced contractions were lower and did not exceed a response of 6 mN. Fitting the data to obtain the ET50 of EFS generated a value of 55.9°C (55.1-55.6°C), and R^2^ of 50%. Additionally, we observed a decrease in the variability of response to EFS at around 58°C. The fit for EFS displays a slightly steeper slope *γ* (-0.58 compared to -0.23 per °C) and a 1°C lower ET50 compared to KPSS-challenged vessels (supplemental Table 2, Fig. 4). Yet, there was no statistically significant difference amongst ET50 of KPSS and EFS (*p*=0.06), nor between the ET50 of methacholine and EFS (*p*=0.37).

### Vasorelaxation

Assessment of the maximal vasorelaxation induced by methacholine was feasible only when U46619 pre-contraction was present. In the majority of the dataset, methacholine resulted in full vasorelaxation at temperatures where U46619 already showed a declined contraction (as observed in Fig. 3 at 50°C and 55°C). As methacholine is endothelium-dependent, this finding suggests that endothelial cells can retain their functionality even after exposure to temperatures around 55°C. The slope *γ* of methacholine-induced endothelial-mediated relaxation was -0.09 per °C, showing a much slower decrease in functionality compared to KPSS-stimulated smooth muscle cells. Methacholine demonstrated an ET50 value of 56.7°C (55.5-58.1°C), with an R^2^ of 48%. There was no statistical significant difference between the ET50 of KPSS and methacholine (*p*=0.82), nor between methacholine and EFS (*p*=0.37).

## Discussion

Our research aimed to evaluate the functionality (capacity to respond to stimuli and contract or relax) of cell types present in and around blood vessels after exposure to hyperthermia (45 - 60°C). In this study we determined the temperature at which significant damage occurs to the endothelial cells, smooth muscle cells, and vascular nerves. The results revealed a decline in the functionality of all three cell types when exposed to temperatures ranging from 55°C to 60°C. Other *in vitro* studies on the effects of hyperthermia seem to agree that cellular damage (measured as loss of ATP, reduced NADH activity, or reduced cell membrane integrity) occurs between 50°C - 60°C [19–24]. Higher temperatures have been reported to cause coagulation, for example, the coagulation temperature for albumin was starting at 65°C [25] and theoretic models for vascular laser therapy have estimated the coagulation temperature to be between 60 to 70°C [26, 27]. It should be noted here that there may be a difference between model calculations and actual temperature, as an *in vitro* study found that laser-induced blood coagulation did not occur until 90 to 100°C [28].

We furthermore calculated ET50 values which were, 56.9°C (CI 56.2-57.7°C), 56.7°C (CI 55.5-58.1°C), and 55.9°C (CI 55.1-55.6°C), for smooth muscle cells, endothelial cells, and perivascular nerves, respectively. Comparing all ET50 fits showed no statistical significance (*p*=0.11) indicating that the ET50 values for the different cell types did not differ significantly, therefore rejecting our hypothesis that there is a difference in the sensitivity of cell types to thermal damage. This suggests that hyperthermia equally affects each cell type in or around a blood vessel. This finding also suggests that the damage that occurs in the skin from selective photothermolysis in PDL treatment is expected to be nonselective for the cell type exposed. However, it is still feasible that there is a cell-type-specific difference in thermal damage *in vivo* because of the distance of a cell type to the absorbing chromophore (hemoglobin, red blood cells). Since endothelial cells are the first to be exposed to hyperthermia, followed by smooth muscle cells and ultimately perivascular nerves, it remains a possibility that perivascular nerves suffer less damage due to a temperature gradient (from the inside of the blood vessel towards the periphery). Whether the temperature gradient after PDL exposure is still within the range of inflicting thermal damage at the location of the perivascular nerves is yet to be established. But given the diameter of the PDL-focused vessels in the papillary dermis of psoriatic patients (± 10 μm) and the surrounding (peri)vascular nerves, which are located at approximately at 5 μm distance from the vessel, it can be reasoned that a temperature gradient at these scales is negligible.

The nonlinear regression analysis as a function of decay of response per increase in °C varied in slope depending on the cell type for which this fit was applied. For example, the slope of EFS stimulation representing nerve functionality was -0.58 per °C, whereas the slope *γ* of KPSS and methacholine representing smooth muscle cells and endothelial cells, respectively, was around –0.23 per °C. A larger slope may indicate that cell death occurs within a narrower temperature range. Future research is needed to confirm whether nerves indeed show a more narrow window of functional deterioration upon hyperthermia than endothelial and/or smooth muscle cells.

To our knowledge, there is no other paper describing the effects of hyperthermia on isolated blood vessels in a wire myography setting. We think that this approach has its merit, as the physiological context of the cell types within the vessel wall is maintained during laser therapy and may impact the results. Yet, other papers on the *in vitro* effects of hyperthermia seem to agree that cellular damage (measured as loss of ATP, reduced NADH activity, or reduced cell membrane integrity) occurs between 50°C-60°C [19–24].

The present study has several limitations. Firstly, regulating heat exposure to the vessels was difficult to control, as it involved changing the medium using a syringe, leading to small variations in temperature application and thus in the vascular response. Secondly, the use of rat mesenteric arteries, although convenient for harvesting, may not fully represent human skin's blood vessels. Investigating more peripheral blood vessels, such as the rat saphenous artery, could be beneficial for future studies in the context of pulsed dye laser treatment that is applied on the skin. Moreover, the experimental design of this study depended on the functionality of the smooth muscle cells, making smooth muscle responses an intrinsic part of the response to the other cell types. Lastly, the response to nerve stimulation could be a mix of contractile and relaxing factors. Thus, nerve-regulated contraction through sympathetic innervation may also be counteracted by sensory nerve signaling. In order to study the function of sensory versus sympathetic nerve functionality separately, an alternative approach offering additional insight might involve a dose-response curve using increasing doses of KPSS. In this manner, within the range of 20–40 mM of K+, one could distinguish which nerve type (sensory or sympathetic) is being activated [29].

## Conclusions

Our data show that the contractile and relaxing functionality of blood vessels significantly decreases when exposed to temperatures between 55-60°C for 30 seconds, with a 50% decrease (ET50) in responsiveness at 56°C. These findings suggests that laser treatment with non-destructive settings can still eliminate blood vessel functionality without inducing coagulation. We also confirmed that functional thermal damage in a blood vessel does not depend on cell type (endothelial cells, smooth muscle cells, and/or perivascular nerves). Additional research is needed to determine the thermal gradient to which nearby tissues are subjected during PDL treatment. This research may advance the understanding of the long-term remission of psoriasis after PDL treatment. Our data on cell viability and cell functionality after hyperthermia may also contribute to other applications of hyperthermia, such as cancer treatment, thermal ablation studies, vascular occlusion, drug delivery, treatment of ischemia and hemostasis.

## Supplementary information

ESM 1 (DOCX 7186 kb)
