# Supplementary material for: The impact of temperature on vascular function in connection with vascular laser treatment
Source: Lasers Med Sci. 2024 May 4;39(1):122. doi: 10.1007/s10103-024-04070-7 (PMC11069475; doi:10.1007/s10103-024-04070-7)
Supplement: Supplementary file 1 — (DOCX 7186 kb) [file 10103_2024_4070_MOESM1_ESM.docx]

Supplements
Supplemental Table 1 constituents of buffers used in wire myography of rat mesenteric arteries.

| **PSS solution** | Model name, brand, manufacturer, city, state, country |  |
| --- | --- | --- |
| 119 mM NaCl | Sigma Aldrich - 71378 | Amsterdam, The Netherlands |
| 4.7 mM KCl | Merck - 49361000 | Merck Chemicals, Amsterdam, The Netherlands |
| 1.18 mM KH_2_PO_4_ | Merck – 1.048730 | Merck Chemicals, Amsterdam, The Netherlands |
| 1.17 mM MgSO_4_ | Merck – 1.05886 | Merck Chemicals, Amsterdam, The Netherlands |
| 0.026 mM EDTA | Sigma Aldrich - ED-500G | Amsterdam, The Netherlands |
| 5 mM HEPES | Merck - 1.101100 | Merck Chemicals, Amsterdam, The Netherlands |
| 25 mM NaHCO_3_ | Merck - 1.06329 | Merck Chemicals, Amsterdam, The Netherlands |
| 1.6 mM CaCl_2_ | Merck - 1.02382 | Merck Chemicals, Amsterdam, The Netherlands |
| 5.6 mM glucose | Merck - 1.08337 | Merck Chemicals, Amsterdam, The Netherlands |
|  |  |  |
| **KPSS solution** |  |  |
| 123.7 mM KCL | Merck - 49361000 | Merck Chemicals, Amsterdam, The Netherlands |
| 1.18 mM KH_2_PO_4_ | Merck – 1.048730 | Merck Chemicals, Amsterdam, The Netherlands |
| 1.17 mM MgSO_4_ | Merck – 1.05886 | Merck Chemicals, Amsterdam, The Netherlands |
| 0.026 mM EDTA | Sigma Aldrich - ED-500G | Amsterdam, The Netherlands |
| 5 mM HEPES | Merck - 1.101100 | Merck Chemicals, Amsterdam, The Netherlands |
| 25 mM NaHCO_3_ | Merck - 1.06329 | Merck Chemicals, Amsterdam, The Netherlands |
| 1.6 mM CaCl_2_ | Merck - 1.06329 | Merck Chemicals, Amsterdam, The Netherlands |
|  |  |  |
| **Ca- free MOPS** |  |  |
| 145 mM NaCl | Sigma Aldrich - 71378 | Amsterdam, The Netherlands |
| 4.7 mM KCl | Merck - 49361000 | Merck Chemicals, Amsterdam, The Netherlands |
| 1.17 mM MgSO_4_ | Merck – 1.05886 | Merck Chemicals, Amsterdam, The Netherlands |
| 0.12 mM NaH_2_ PO_4_ | Merck - 1.06346 | Merck Chemicals, Amsterdam, The Netherlands |
| 3.0 mM MOPS | Sigma Aldrich - M1254 | Amsterdam, The Netherlands |
| 5.04 mM Glucose | Merck - 1.08337 | Merck Chemicals, Amsterdam, The Netherlands |
| 2.52 mM Pyruvate | Sigma - P2256 | Amsterdam, The Netherlands |


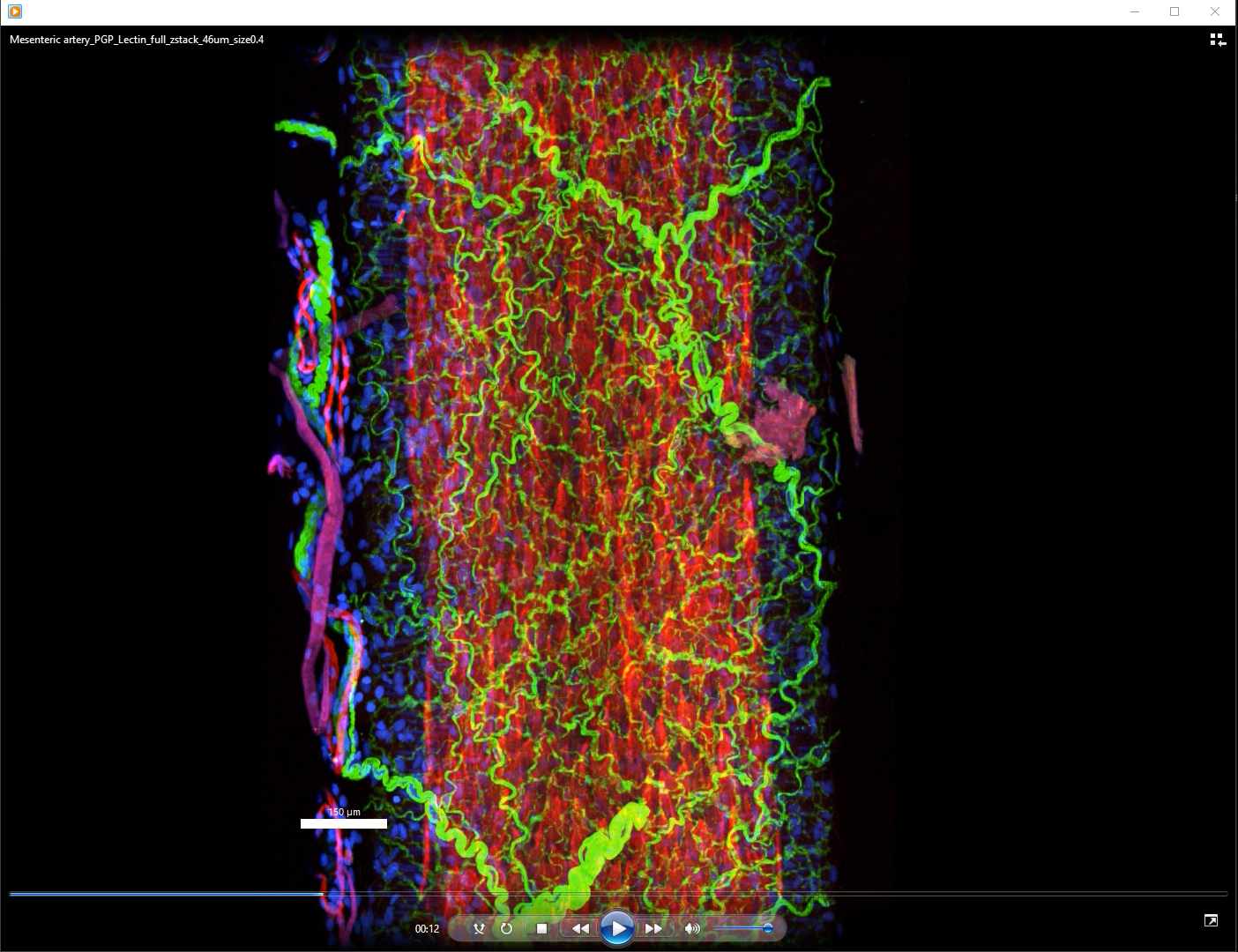


Supplemental Video 1 3D video of blood vessel in IMARIS [Mesenteric artery - YouTube](https://www.youtube.com/watch?v=-xcdQabHq3A). If the link does not work then please copy/paste: <https://www.youtube.com/watch?v=-xcdQabHq3A>


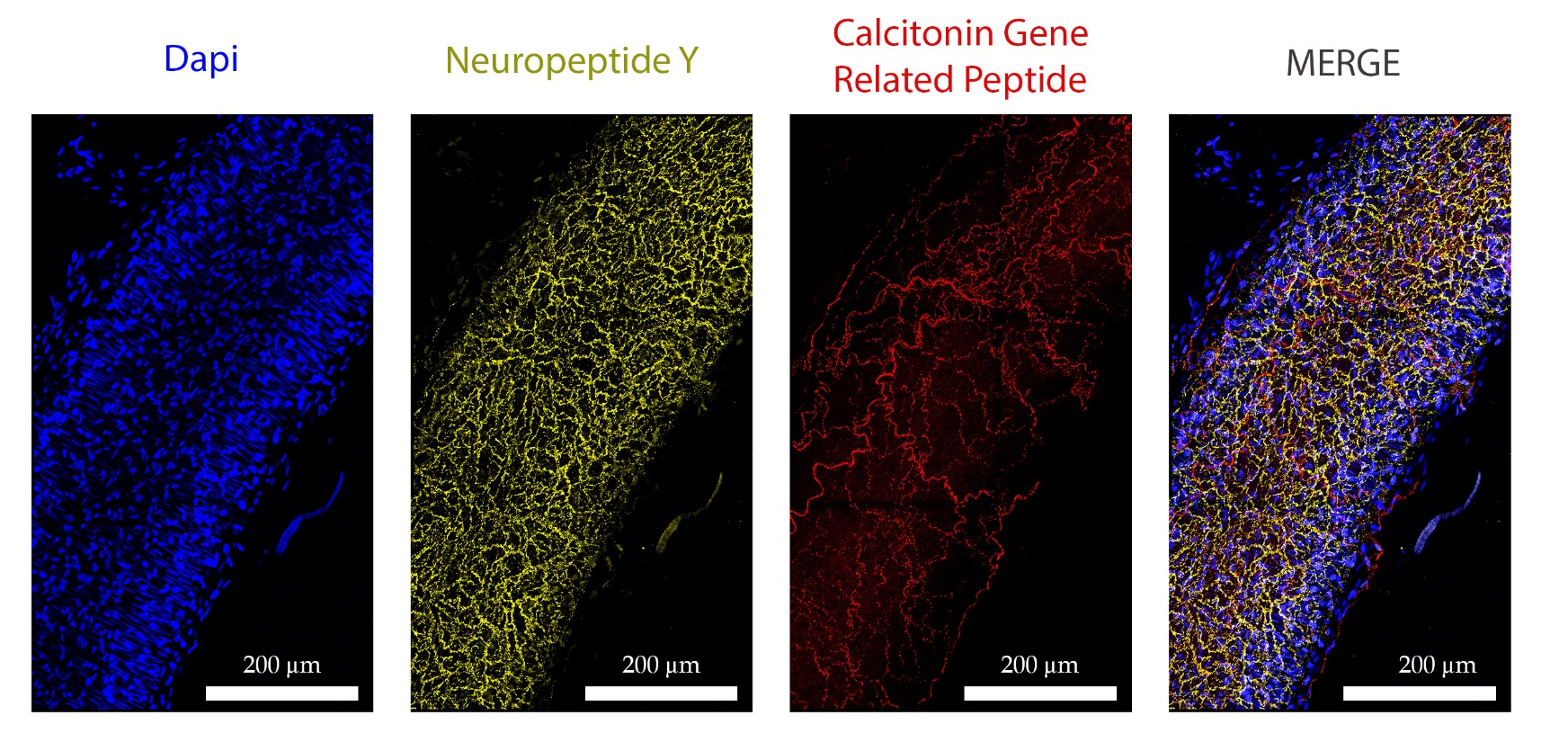


Supplemental Fig. 1 Neuropeptide Y (NPY) and Calcitonin Gene Related Peptide (CGRP) stained segment of rat mesenteric artery. Left: Dapi staining to indicate the nuclei. NPY staining in yellow to indicate the sympathetic nerve fibers, CGRP in red to indicate the sensoric nerves within the adventitia of the blood vessel. Right panel: merge of DAPI, NPY, and CGRP, showing the innervation of rat mesenteric artery segment. Scale bar indicates 200 µm


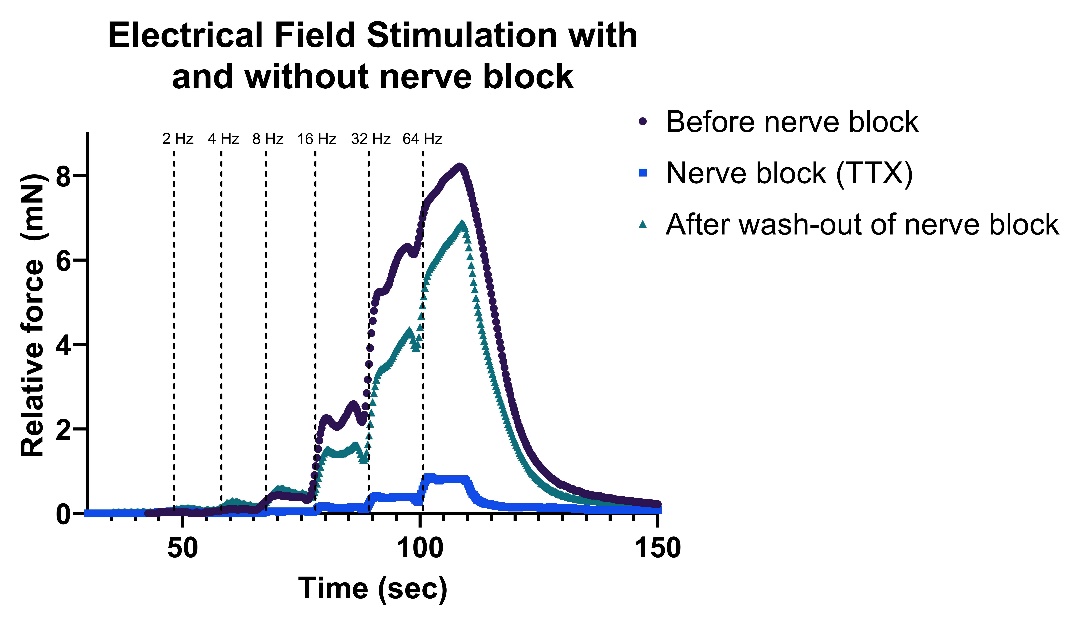


Supplemental Fig. 2 Tetrodotoxin (TTX, 3 µM) nerve-blocking abolishes contraction upon electrical field stimulation. The graph shows how TTX could abolish contraction up to 16 Hz, whereas a non-nerve regulated contraction could still be seen at 32 Hz and 64 Hz

Supplemental Table 2 descriptive results for the non-linear regression fit. Bottom values were constrained to 0 and are therefore not shown

|  | KPSS | U46619 | Methacholine | Electrical Stimulation |
| --- | --- | --- | --- | --- |
| Top | 1.13 | 0.78 | 0.93 | 0.89 |
| Slope (per °C ,$\gamma$) | -0.23 | -0.09 | -0.22 | -0.58 |
| ET50 (°C) | 56.9 | 51.4 | 56.7 | 55.9 |
| 95% C.I. | 56.2 to 57.7 | 47.0 to 55.8 | 55.3 to 58.2 | 55.1 to 56.7 |
| Goodness of fit (R^2^ ) | 0.67 | 0.34 | 0.48 | 0.50 |
